# Effect of preoperative pulse oximeter oxygen saturation on postoperative prolonged mechanical ventilation in patients with tetralogy of Fallot

**DOI:** 10.3389/fcvm.2022.967240

**Published:** 2022-08-22

**Authors:** Xie Wu, Ran An, Qipeng Luo, Yinan Li, Hongbai Wang, Qiao Liu, Jiangshan Huang, Yuan Jia, Su Yuan, Fuxia Yan

**Affiliations:** Department of Anesthesiology, National Center for Cardiovascular Diseases, Fuwai Hospital, Chinese Academy of Medical Sciences and Peking Union Medical College, Beijing, China

**Keywords:** pulse oximeter oxygen saturation (SpO_2_), tetralogy of Fallot (TOF), prolonged mechanical ventilation (PMV), risk factor, prognosis

## Abstract

**Background:**

As an easily accessible and intervened clinical indicator, preoperative pulse oximeter oxygen saturation (SpO_2_) is an important factor affecting the prognosis of patients with tetralogy of Fallot (TOF). However, whether SpO_2_ is associated with postoperative mechanical ventilation (MV) time remains unknown. Therefore, this study aimed to investigate the impact of preoperative SpO_2_ on postoperative prolonged mechanical ventilation (PMV) in children with TOF.

**Materials and methods:**

The study included children younger than 18 years who underwent corrective operations for TOF between January 2016 and December 2018 in Fuwai Hospital, China. Univariate and multivariate logistic regression analyses were used to evaluate the influence of preoperative SpO_2_ on postoperative PMV. After identifying SpO_2_ as an independent risk factor for PMV, patients were further divided into two groups according to the cutoff value of SpO_2_, and propensity score matching (PSM) analysis was used to eliminate the effect of confounding factors. The logistic regression was used to compare the outcomes between the two groups after PSM.

**Results:**

A total of 617 patients were finally enrolled in this study. By the univariable and multivariate logistic analysis, four independent risk factors for PMV were determined, namely, SpO_2_, surgical technique, aortic cross-clamp time, and intraoperative minimum temperature. According to the outcomes of 219 paired patients after PSM, the incidence of PMV was significantly higher in patients with lower preoperative SpO_2_ (*P* = 0.022). Also, there was significant increase in mechanical ventilation time (*P* = 0.019), length of intensive care unit stay (*P* = 0.044), postoperative hospital stay (*P* = 0.006), hospital stay (*P* = 0.039), and hospitalization cost (*P* = 0.019) at the lower preoperative SpO_2_ level.

**Conclusion:**

Low preoperative SpO_2_ represents an independent risk factor of postoperative PMV in children with TOF.

## Introduction

Tetralogy of Fallot (TOF) is one of the most common forms of cyanotic congenital heart disease (1), with an estimated incidence of 3/10000 worldwide (2). At present, complete repair of TOF is the preferred treatment. Over the past decades, long-term outcomes of patients with TOF have improved a lot due to the advances in surgical techniques (3, 4). Therefore, most studies in recent years began to focus on the short-term outcomes of TOF patients. However, due to the existence of intracardiac shunt, most of the TOF patients present with cyanosis, dyspnea and chronic hypoxia before surgery (5). In addition, the repair of TOF requires longer cardiopulmonary bypass (CPB) and operating time because of the complex cardiac malformations. Therefore, patients with TOF are more likely to develop pulmonary injury after surgery (6).

The mechanical ventilation (MV) time is correlated with the pulmonary function recovery after anesthesia and surgery. Previous studies have demonstrated that prolonged mechanical ventilation (PMV) was associated with adverse outcomes (7, 8). Early extubation in adults after cardiac surgery has been shown to reduce intensive care unit (ICU) stay, lead to lower hospitalization costs and have better prognosis (9). However, risk factors for PMV and the effect of PMV on prognosis in children with TOF have not been fully investigated.

As an easily accessible and intervened perioperative indicator, pulse oximeter oxygen saturation (SpO_2_) is closely related to human respiratory and circulatory physiology (10, 11). Previous studies have shown that the proportion of time spent in different degrees of SpO_2_ is linked to hospital mortality in an ICU population with MV (12, 13). Nevertheless, few studies have considered the prognostic impact of SpO_2_ for children following TOF repair. In this retrospective study, we aimed to evaluate the association of preoperative SpO_2_ with PMV in children with TOF.

## Materials and methods

### Study design and participants

A total of 782 children (age <18) undergoing TOF repair at Fuwai Hospital (National Center for Cardiovascular Diseases, Beijing, China) were enrolled in this observational retrospective study (January 2016 to December 2018). The study was approved by the Institutional Review Board, and informed consent was waived.

**FIGURE 1:**
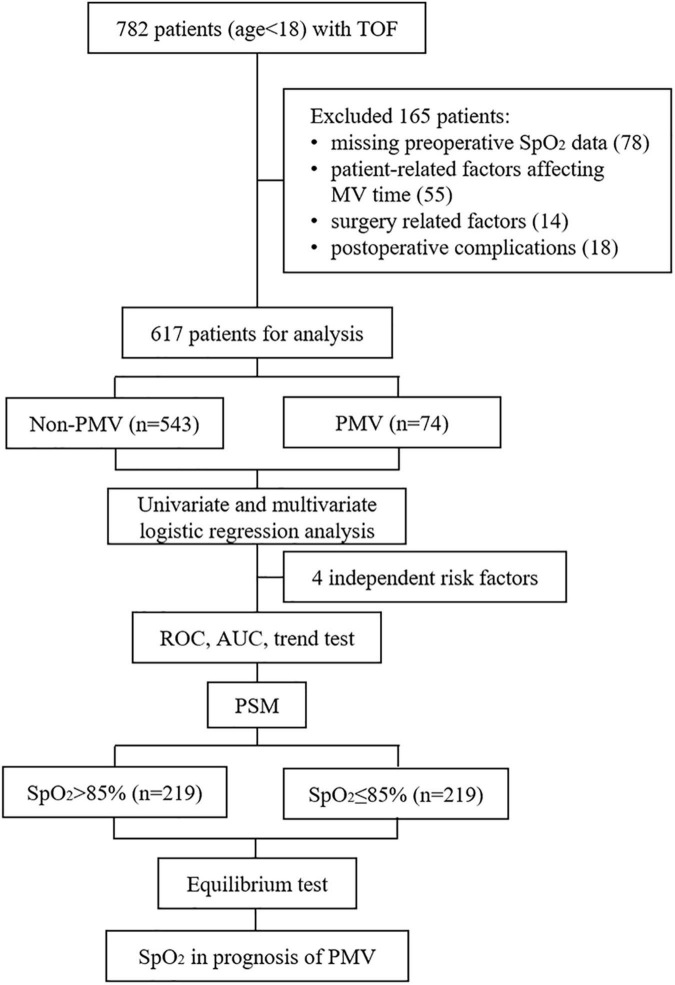
Flowchart of this study. TOF, Tetralogy of Fallot; SpO_2_, pulse oximeter oxygen saturation; MV, mechanical ventilation; PMV, prolonged mechanical ventilation; ROC, receiver operating characteristic; AUC, area under the curve; PSM, propensity score matching.

The exclusion criteria for participants included: (1) patients with missing data on preoperative SpO_2_; (2) patient related factors that may affect the MV time, including smoking history, previous palliative surgery, preoperative endotracheal intubation or laryngeal mask airway, concurrent complex malformations (double-outlet right ventricle, complete endocardial cushion defect, and severe pulmonary hypertension), and genetic syndrome; (3) surgery related factors including emergency surgery, second surgery, and bedside thoracotomy; (4) postoperative complications including postoperative death, arrhythmia, thrombosis, and the need for peritoneal dialysis, tracheotomy or extracorporeal membrane oxygenation. As shown in Figure 1, after excluding the patients with SpO_2_ absence or specific factors affecting postoperative MV time, 617 subjects were finally included in the statistical analysis.

### Surgical technique

In TOF repair of all patients, the infundibulum was incised longitudinally to just below the level of the pulmonary valve annulus under CPB and moderate hypothermia. Hypertrophied right ventricular parietal muscle bundles were resected, and the ventricular septal defect was closed with a pericardial patch. All subjects received either pulmonary valve-sparing (VS) repair or transannular patch (TAP) repair with right ventricular outflow tract incision. VS repair was defined as uncut pulmonary valve. The choice of VS or TAP repair depends not only on the patient’s individual anatomy, but also on the surgeon’s own preference and practice mode.

### Variable measurement and outcomes

The medical records of all patients recruited were retrospectively reviewed, and the following perioperative information was collected. Demographic data included gestational age, age at operation, sex, weight, height, and body surface area (BSA). BSA was calculated as follows: BSA = 0.0061 × height (cm) + 0.0128 × weight (kg) – 0.1529. Preoperative data included SpO_2_, American Society of Anesthesiologists Physical Status Classification, left ventricular ejection fraction, major aortopulmonary collateral arteries (MAPCAs) and routine blood test results, including the count of white blood cell, absolute lymphocyte (ALC) and platelet (PLT), and the level of blood urea nitrogen (BUN), creatinine (Cr), and albumin (Alb). Intraoperative data included CPB time, aortic cross-clamp (ACC) time, the minimum temperature (T_min_), blood loss, and infusion volume.

The primary outcome was PMV, which is defined as MV support time ≥48 h.

Mechanical ventilation was considered to include only tracheal intubation, and non-invasive ventilation such as CPAP or BiPAP was not considered as MV in this study. Secondary outcome measures included MV time, length of ICU stay, length of postoperative hospital stay, length of hospital stay, and hospitalization cost.

### Statistical analysis

Continuous variables were reported as median and interquartile range, and categorical variables were presented as count and proportion. The differences in baseline characteristics and clinical variables between non-PMV and PMV groups were evaluated using the Mann–Whitney *U* test for continuous variables and Chi-squared test for categorical variables. Univariate and multivariate logistic regression analyses were used to evaluate the influence of preoperative SpO_2_ on postoperative PMV. In the multivariate analysis, we adjusted the variables with statistical significance (*P* < 0.1) or recognized as clinically significant in the univariate analysis, including age, BSA, preoperative SpO_2_, MAPCAs, PLT, BUN, Alb, surgical technique, CPB time, ACC time, T_min_, and blood loss. The results were presented with odds ratios (ORs) and 95% confidence intervals (CIs).

After SpO_2_ was identified as an independent risk factor for PMV, we then calculated the cut-off value of SpO_2_ through receiver operating characteristic (ROC) curve and area under the curve (AUC). Furthermore, the subjects were also equally divided into five groups based on the SpO_2_ level, and *P* value for linear trend was calculated for SpO_2_ and the incidence of PMV.

Patients were further divided into SpO_2_ >85% and SpO_2_ ≤85% groups according to the cutoff value, and propensity score matching (PSM) analysis was used to eliminate the effect of confounding factors, including age, BSA, MAPCAs, PLT, BUN, Alb, surgical technique, CPB time, ACC time, T_min_, and blood loss. Through nearest neighbor matching, each patient in the SpO_2_ >85% group was matched with one patient in the SpO_2_ ≤85% group, with a caliper width of 0.02. The logistic regression was used to compare the outcomes between the two groups after PSM.

STATA 15.1 Software (StataCorp Stata MP 15.1, United States) for Windows was used for all analyses. Statistical significance was set at a *P* of <0.05 (two-tailed).

## Results

### Characteristics of patients

A total of 617 patients were finally enrolled in this study, including 74 patients with PMV and 543 patients without PMV. The baseline and clinical characteristics of the patients in both groups are shown in Table 1. Compared with the non-PMV group, the patients with PMV were significantly younger (*P* < 0.001), and had significantly lower BSA (*P* < 0.001), preoperative SpO_2_ (*P* < 0.001), BUN (*P* < 0.001) and ALB (*P* = 0.026), and higher MAPCAs (*P* = 0.038). For surgical technique, patients underwent transannular patch were more likely to have PMV compared to who underwent valve sparing procedure (*P* < 0.001). During the procedures, the patients with PMV had significantly longer CPB (*P* < 0.001) and ACC time (*P* < 0.001), and lower T_min_ (*P* < 0.001).

**TABLE 1:** The demographic and perioperative data in patients with and without PMV.

Variables	Non-PMV (*n* = 543)	PMV (*n* = 74)	*P*
**Demographics**			
Gestational age (month)	39 (38, 40)	39 (37.5, 40)	0.973
Age at operation (month)	10 (7, 15)	7 (6, 9.25)	<0.001
Sex (M/F)	331/212	43/31	0.638
Weight (kg)	9 (8, 10)	8 (7, 9)	<0.001
Height (cm)	71 (67, 78)	66.5 (64, 70)	<0.001
BSA (m^2^)	0.39 (0.35, 0.45)	0.35 (0.33, 0.39)	<0.001
**Preoperative data**			
SpO_2_ (%)	89 (82, 95)	84 (74.5, 90.5)	<0.001
ASA class (III/IV)	(491/52)	(66/8)	0.878
LVEF (%)	66 (64, 71)	67 (63, 71.1)	0.966
MAPCAs (n, %)	75 (13.8%)	17 (23.0%)	0.038
Blood test			
WCC (10^9^/L)	10.41 (8.32, 12.93)	10.48 (8.47, 13.02)	0.821
ALC (10^9^/L)	6.83 (5.07, 9.09)	7.15 (5.50, 9.42)	0.613
PLT (10^9^/L)	322 (258, 390)	290 (205, 390)	0.099
BUN (mmol/L)	3.34 (2.37, 4.32)	2.56 (1.90, 3.48)	<0.001
Cr (μmol/L)	30 (26, 34)	29 (25, 33)	0.315
Alb (g/L)	44.7 (42.7, 46.3)	44.0 (41.1, 46.1)	0.026
**Intraoperative data**			
Surgical technique (TAP/VS)	96 (17.7%)/447 (82.3%)	26 (35.1%)/48 (64.9%)	<0.001
CPB time (min)	95 (80, 116.5)	116.5 (96.75, 131)	<0.001
ACC time (min)	66 (54, 83)	78.5 (63, 100.5)	<0.001
T_min_ (°C)	30 (29, 30)	28 (27, 29)	<0.001
Blood loss (ml)	30 (20, 40)	30 (25, 50)	0.070
Infusion volume (ml)	60 (45, 80)	60 (45, 80)	0.806
MV time (h)	16 (8, 24)	22 (13, 43.75)	<0.001

Data expressed as number of patients (%), or median (interquartile intervals). BSA, body surface area; SpO_2_, pulse oximeter oxygen saturation; ASA class, American Society of Anesthesiologists Physical Status Classification; LVEF, left ventricular ejection fraction; MAPCAs, major aortopulmonary collateral arteries; WCC, white blood cell count; ALC, absolute lymphocyte count; PLT, platelet counts; BUN, blood urea nitrogen; Cr, creatinine; Alb, albumin; TAP, transannular patch; VS, valve sparing procedure; CPB, cardiopulmonary bypass; ACC, aortic cross-clamp; T_min_, the minimum temperature; MV, mechanical ventilation.

### Independent predictors for prolonged mechanical ventilation

Based on univariate analysis, 12 factors were selected for multivariate analysis. On multivariate analysis, 4 independent risk factors for PMV were determined, namely, SpO_2_ (OR 0.969; 95% CI 0.948–0.990; *P* = 0.004), VS procedure (OR 0.484; 95% CI 0.263–0.891; *P* = 0.020), ACC time (OR 1.018; 95% CI 1.006–1.030; *P* = 0.003), and T_min_ (OR 0.777; 95% CI 0.670–0.902; *P* = 0.001) (Table 2).

**TABLE 2:** Univariable and multivariable logistic regression analyses of potential risk factors in the study population.

Variables	Univariable analysis		Multivariable analysis	
	OR (95% CI)	*P*	OR (95% CI)	*P*
Age (month)	0.878 (0.824, 0.935)	<0.001		
BSA (m^2^)	0.000 (0.000, 0.002)	<0.001		
SpO_2_ (%)	0.966 (0.947, 0.984)	<0.001	0.969 (0.948, 0.990)	0.004
MAPCAs (n, %)	1.861 (1.028, 3.371)	0.040		
PLT (10^9^/L)	0.998 (0.996, 1.000)	0.113		
BUN (mmol/L)	0.816 (0.674, 0.989)	0.038		
Alb (g/L)	0.890 (0.820, 0.966)	0.005		
Surgical technique				
TAP	1		1	
VS	0.396 (0.234, 0.671)	<0.001	0.484 (0.263, 0.891)	0.020
CPB time (min)	1.005 (0.999, 1.011)	0.101		
ACC time (min)	1.021 (1.012, 1.030)	<0.001	1.018 (1.006, 1.030)	0.003
T_min_ (°C)	0.683 (0.596, 0.783)	<0.001	0.777 (0.670, 0.902)	0.001
Blood loss (ml)	1.000 (0.994, 1.006)	0.948		

OR, odds ratio; CI, confidence interval; BSA, body surface area; SpO_2_, pulse oximeter oxygen saturation; MAPCAs, major aortopulmonary collateral arteries; PLT, platelet counts; BUN, blood urea nitrogen; TAP, transannular patch; VS, valve sparing procedure; CPB, cardiopulmonary bypass; ACC, aortic cross-clamp; T_min_, the minimum temperature.

### Cutoff value of SpO_2_

**FIGURE 2:**
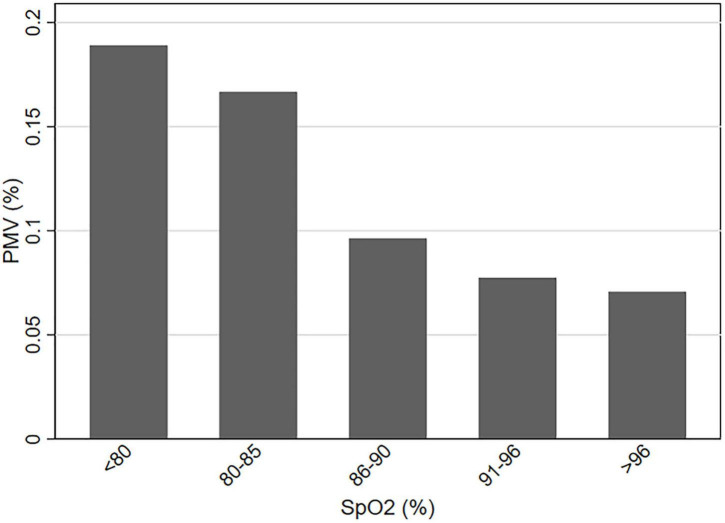
The incidence of PMV at different levels of preoperative SpO_2_. PMV, prolonged mechanical ventilation; SpO_2_, pulse oximeter oxygen saturation.

Receiver operating characteristic curve was used to evaluate the predictive performance of the multi-factor model constructed by preoperative SpO_2_, surgical technique, ACC time, and T_min_ for postoperative PMV, and the results showed that the model had good prediction performance, with an AUC of 0.838 (95% CI, 0.794–0.882). The cut-off value of preoperative SpO_2_ was calculated by ROC curve to be 85% (AUC, 0.640; 95% CI, 0.570–0.709). Then, the subjects were equally divided into five groups according to preoperative SpO_2_ level, and the incidence of postoperative PMV in each group was calculated (Figure 2). The results exposed that the incidence of postoperative PMV showed a clear decrease as preoperative SpO_2_ increased (*P* < 0.01), and bounded by 85%, the incidence of PMV decreased significantly in patients with preoperative SpO_2_ over 85%.

### Baseline characteristics of subjects according to the cut-off value of SpO_2_

**FIGURE 3:**
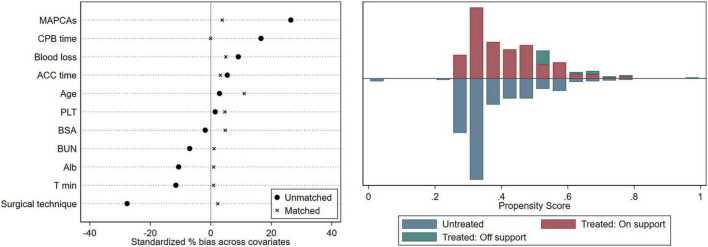
Equilibrium test. CPB, cardiopulmonary bypass; ACC, aortic cross-clamp; PLT, platelet; BSA, body surface area; BUN, blood urea nitrogen; ALB, albumin; T_min_, the minimum temperature.

Demographic and clinical variables, according to the optimal cutoff value of SpO_2_, are shown in Table 3. Subjects were classified into two groups: SpO_2_ >85% group (*n* = 376) and SpO_2_ ≤85% group (*n* = 241). Since a variety of variables were imbalanced between the two groups of patients, we implemented a 1:1 PSM to reduce potential confounding effects. In the PSM analysis, we obtained 219 patients from SpO_2_ >85% group with matched pairs of 219 patients whose SpO_2_ ≤85%, the results were shown in Table 3. The demographic data and clinical characteristics were well-balanced and evenly distributed between the two groups after PSM (Standardized mean difference <0.10) (Figure 3).

**TABLE 3:** The demographic and perioperative data before and after PSM.

Variables SpO_2_ (%)	All patients (*n* = 617)			Matched patients (*n* = 438)		
	>85% (*n* = 376)	≤85% (*n* = 241)	*P*	>85% (*n* = 219)	≤85% (*n* = 219)	*P*
Age (month)	9 (7, 14)	9 (6, 15)	0.862	9 (6, 13)	9 (6, 15)	0.508
BSA (m^2^)	0.39 (0.35, 0.44)	0.39 (0.34, 0.44)	0.552	0.39 (0.35, 0.44)	0.39 (0.35, 0.43)	0.902
MAPCAs (n, %)	42 (11.17%)	50 (20.75%)	0.001	38 (17.35%)	41 (18.92%)	0.709
PLT (10^9^/L)	321 (254, 385)	322 (249, 401)	0.872	319 (240, 381)	322 (253, 401)	0.503
BUN (mmol/L)	3.2 (2.3, 4.3)	3.2 (2.3, 4.1)	0.530	3.3 (2.3, 4.3)	3.3 (2.3, 4.2)	0.620
Alb (g/L)	44.6 (42.4, 46.2)	44.8 (42.7, 46.5)	0.721	44.2 (42.4, 45.9)	44.8 (42.8, 46.5)	0.165
Surgical technique (TAP/VS)	(58/318)	(64/177)	0.001	(55/164)	(53/166)	0.825
CPB time (min)	95 (80, 118)	100.5 (86, 123)	0.009	101 (85, 122)	100 (86, 123)	0.712
ACC time (min)	67 (54, 85)	68 (56, 84)	0.406	68 (54, 86)	69 (57, 85)	0.576
T_min_ (°C)	30 (29, 30)	29 (28, 30)	0.020	30 (28, 30)	30 (28, 30)	0.668
Blood loss (ml)	30 (20, 40)	30 (20, 45)	0.196	30 (20, 50)	30 (20, 45)	0.703

Data expressed as number of patients (%), or median (interquartile intervals). PSM, propensity score matching; SpO_2_, pulse oximeter oxygen saturation; BSA, body surface area; MAPCAs, major aortopulmonary collateral arteries; PLT, platelet counts; BUN, blood urea nitrogen; Alb, albumin; CPB, cardiopulmonary bypass; ACC, aortic cross-clamp; T_min_, the minimum temperature.

### The outcomes of the patients

The adjusted cohort after PSM was then used to compare the PMV and the secondary outcomes. There was a significant increase in the incidence of postoperative PMV depending on the lower SpO_2_ level: SpO_2_ >85% group, 20 (9.13%); SpO_2_ ≤85% group, 36 (16.44%) (*P* = 0.022). Furthermore, as depicted in Table 4, patients with lower SpO_2_ level had significantly higher hospitalization cost (*P* = 0.019), and longer periods of MV time (*P* = 0.019), ICU stay (*P* = 0.044), postoperative hospital stay (*P* = 0.006), and hospital stay (*P* = 0.039). One point to note in Table 4 is that 0 h of MV time means the patients were extubated in the operating room due to ultra-fast-track anesthesia.

**TABLE 4:** Outcomes of the patients with higher and lower SpO_2_ levels after PSM.

Outcome	SpO_2_		*P*
	>85% (*n* = 219)	≤85% (*n* = 219)	
PMV	20 (9.13%)	36 (16.44%)	0.022
MV time (h)	18 (9, 28); (0, 121)	21 (12, 34); (0, 223)	0.019
LOIS (d)	2 (1, 4); (1, 14)	3 (1, 4); (1, 65)	0.044
LOHS (d)	14 (12, 17); (7, 48)	15 (13, 20); (8, 80)	0.039
LOPS (d)	8 (7, 11); (5, 30)	10 (7, 12); (5, 72)	0.006
Cost (¥ 1000)	70 (61, 83); (43, 194)	74 (65, 89); (7, 554)	0.019

Data expressed as number of patients (%), or median (interquartile intervals); (complete ranges). PSM, propensity score matching; SpO_2_, pulse oximeter oxygen saturation; PMV, prolonged mechanical ventilation; MV, mechanical ventilation; LOIS, length of ICU stay; LOHS, length of hospital stay; LOPS, length of postoperative hospital stay.

In addition, since the main purpose of this study was to investigate the influence of preoperative SpO_2_ on postoperative PMV, patients pronounced dead and with other complications that might affect MV time were excluded. To further explore the effect of preoperative SpO_2_ on mortality and morbidity of complications, all patients were selected for analysis. As described in Table 5, patients with lower SpO_2_ level had significantly higher rates of in-hospital mortality (*P* = 0.048), ECMO placement (*P* = 0.025), re-ICU (*P* = 0.004), re-intubation (*P* = 0.019), thromboembolic events (*P* = 0.032), significant cardiac disorders (*P* = 0.004) and acute kidney injury (*P* = 0.001), with statistical significance.

**TABLE 5:** Mortality and morbidity of the patients based on preoperative SpO_2_.

Outcome	SpO_2_		*P*
	>85% (*n* = 490)	≤85% (*n* = 292)	
In-hospital mortality	1 (0.20%)	4 (1.37%)	0.048
ECMO placement	0	3 (1.03%)	0.025
Re-ICU	15 (3.06%)	22 (7.53%)	0.004
Re-intubation	28 (5.71%)	30 (10.27%)	0.019
Thromboembolic events	3 (0.61%)	7 (2.40%)	0.032
Significant cardiac disorders	4 (0.82%)	11 (3.77%)	0.004
Chest drainage time (d)	3 (2, 4); (1, 17)	3 (2, 4); (2, 11)	0.861
Acute kidney injury	52 (10.61%)	55 (18.84%)	0.001

Data expressed as number of patients (%), or median (interquartile intervals); (complete ranges). SpO_2_, pulse oximeter oxygen saturation; ECMO, extracorporeal membrane oxygenation; ICU, intensive care unit.

## Discussion

Mechanical ventilation time is an important factor determining the recovery and prognosis of patients after surgery, as well as a significant indicator reflecting lung injury in TOF patients. Most of the previous research has mainly focused on the prognostic significance of PMV (14), but less on the risk factors that may lead to PMV. The limited data on MV time in TOF patients are available just from studies with a small number of subjects or patients with mixed diagnosis (9, 15).

Tetralogy of Fallot patients are often in a long-term chronic hypoxic condition which causes damage to some vital organs including the lung (16, 17). In addition, due to complex cardiac malformations, there seems to be a longer period of procedural and CPB time in the repair of TOF, and higher incidence of postoperative PMV and acute lung injury (ALI) (18). SpO_2_ is an easily accessible and common perioperative clinical indicator. Although a small number of studies have demonstrated the value of SpO_2_ in preoperative evaluation and long-term prognosis of patients with TOF (19, 20), whether SpO_2_ is associated with postoperative MV time remains unknown.

Thus, we carried out the study focusing on this issue. In this study, we retrospectively compared the preoperative and intraoperative data that may affect the postoperative MV time of TOF patients, confirmed that SpO_2_ was an independent risk factor for PMV through univariate and multivariate logistic regression, and determined that the cutoff value of SpO_2_ was 85% by calculating the area under the receiver operating characteristic curve. Then the patients were regrouped according to the level of preoperative SpO_2_ (SpO_2_ >85% or SpO_2_ ≤85%), and confounding factors were controlled by PSM. Finally, the results showed that there was a significantly higher incidence of postoperative PMV depending on the lower preoperative SpO_2_ level, and the optimal cut-off value of SpO_2_ was 85%. Patients with preoperative SpO_2_ ≤85% had significantly longer periods of MV time, length of ICU stay, length of postoperative hospital stay, and length of hospital stay, and higher hospitalization cost. Beyond, it was shown that surgical technique, intraoperative ACC time and T_min_ were also the independent risk factors for PMV in children following TOF repair.

In our clinic, we found that the postoperative MV time of patients with cyanosis congenital heart diseases was usually longer than that of non-cyanosis patients. Except for more complex cardiac malformations and more difficult and longer surgeries, is PMV related to long-term chronic hypoxia before surgery in patients with cyanosis? To explore this question, we designed the study. In order to reduce the influence of different types of diseases and different degrees of heart malformations on the conclusion, we only enrolled patients with TOF, and excluded that with complex malformations. We found that patients with lower preoperative SpO_2_ had significantly longer postoperative MV time. However, the specific mechanisms for this phenomenon remain unclear, and this will be a direction of our future work.

Based on the previous research, we speculated and proposed some possible mechanisms. The repair of TOF is performed with CPB and cardiac arrest. During cardiac arrest, the blood only from bronchial arteries is far from sufficient to meet the metabolic needs of the lung, and the lung is in a state of severe ischemia and hypoxia. Therefore, postoperative lung ischemia-reperfusion injury (LIRI) is inevitable (21). Patients with cyanosis tend to have more complex heart malformations and longer CPB time, which results in more severe LIRI after surgery (6). Moreover, preoperative long-term chronic hypoxia may lead to some compensatory changes in lung tissue, mainly manifested as pulmonary vascular remodeling and hyperplasia (22). Although neovascularization can improve the oxygenation of patients, it reduces the self-protection ability of lung tissue against the surgical stress, represented mainly by increasing pulmonary vascular permeability, more serious pulmonary edema, and the occurrence of sterile inflammatory reaction of the lung after CPB and LIRI. Because of the interaction of all the above factors, patients with low preoperative oxygenation tend to have more severe LIRI after surgery, which may require longer recovery and MV time.

In this research, MV time was used as a surrogate of postoperative lung injury, which was usually assessed in terms of severity of acute respiratory distress syndrome (ARDS) in other studies. The Berlin Conference definition of ARDS (23) is usually not applicable in children due to the difference in ventilatory strategy used in pediatric patients. Commonly used parameter to grade the severity of pediatric ARDS is oxygenation index [OI, OI = (FiO_2_ × Paw × 100)/PaO_2_] (24, 25). In recent years, improvement in CPB technology has increased the safety of surgery for congenital heart diseases; still, postoperative ARDS remains a life-threatening complication after cardiac surgery in children (26–28). According to domestic and international research, incidence of ARDS in pediatric intensive care unit is 1–4%, and the mortality rate is as high as 22–65% (29). Due to retrospective nature of the study, we had difficulty in extracting the data for incidence of postoperative ARDS in our patients. Further, several children with TOF who failed to meet the diagnostic criteria for ARDS after surgery also had long postoperative MV time due to LIRI (data not included in the manuscript). Therefore, we believe that compared with ARDS, MV time is more reasonable indicator for evaluating lung injury keeping in mind that some patients may require prolonged ventilation due to non-pulmonary causes. We plan to investigate the incidence of pediatric ARDS and MV time simultaneously in future prospective studies, so as to evaluate the lung injury after TOF repair more comprehensively.

The strengths of the study included a large sample size, high reliability and rigorous scientific design and approach. In this study, patients were first divided into PMV and non-PMV groups for a case-control study to find all possible risk factors, determine the cut-off value of SpO_2_ and verify the value through multiple methods. Then, in order to prevent potential reverse causation, patients were regrouped according to preoperative SpO_2_ for cohort study and PSM was used to reduce the influence of confounding factors. Eventually, we obtained the same results through a variety of different methods, which enhanced the creditability of our conclusions.

Meanwhile, there were also some limitations to this study. First, the retrospective nature of the study might have led to the missing of important data. Some patients were excluded due to the lack of preoperative SpO_2_, and we were unable to fully review the patients’ conditions for accurate diagnosis of ARDS. We also had no data on pulmonary regurgitation associated with postoperative PMV, which may impose certain biases to the results. Second, this was a single-center study, which may have introduced selection bias. What’s more, age of the subjects at surgery may also influence the generalization of the conclusion. More importantly, the level of preoperative SpO_2_ may be affected by the degree of cardiac malformations. Although in order to minimize the impact of heart malformations on prognosis, only TOF patients were enrolled to this study and patients with complex malformations were excluded, there was no denying the fact that non-randomized experiment had its own defects. It should be noted that the analysis of mortality and morbidity in Table 5 is only an exploratory analysis without strict inclusion and exclusion criteria, and control for confounding factors. Therefore, further more scientifically rigorous multi-center prospective studies are needed to validate our findings.

## Conclusion

As an important indicator of patients’ preoperative oxygenation status, SpO_2_ had a strong predictive value for the occurrence of postoperative PMV, and may help us to early identify TOF patients at high risk of postoperative lung injury. Patients with lower preoperative SpO_2_ had higher incidence of PMV, longer postoperative MV time, ICU stay, postoperative hospital stay, and total hospital stay, and more hospitalization costs. Further prospective studies are needed to determine the ability of SpO_2_ to guide perioperative management in patients undergoing the repair of TOF.

## Data availability statement

The datasets presented in this article are not readily available because other related projects are in progress. Requests to access the datasets should be directed to the corresponding author.

## Ethics statement

The studies involving human participants were reviewed and approved by the Fuwai Hospital. Written informed consent from the participants’ legal guardian/next of kin was not required to participate in this study in accordance with the national legislation and the institutional requirements.

## Author contributions

XW, RA, and FY were involved in the design of the study. XW, YL, HW, QLi, and JH helped in acquisition of data. XW and RA contributed to select, analyze, and interpret the data. XW, RA, YJ, and SY were involved in drafting and final revision of the manuscript. FY helped in study supervision. All authors read and approved the submitted version.
